# Flame-Retardant Performance of Transparent and Tensile-Strength-Enhanced Epoxy Resins

**DOI:** 10.3390/polym12020317

**Published:** 2020-02-04

**Authors:** Liang Li, Zaisheng Cai

**Affiliations:** 1 College of Chemistry, Chemical Engineering and Biotechnology, Donghua University, Shanghai 201620, China; liliang1618@126.com; 2 School of Pharmacy, Yancheng Polytechnic College, Yancheng 224005, China

**Keywords:** thermosets, amine curing agent, DOPO derivatives, mechanical properties, epoxy resins

## Abstract

In this study, a flame-retardant additive with 9,10-dihydro-9-oxa-10-phosphaphenanthrene-10-oxide (DOPO) groups denoted DSD was successfully synthesized from DOPO, 4,4′-diaminodiphenyl sulfone (DDS), and salicylaldehyde. The chemical structure of DSD was characterized by FTIR–ATR, NMR, and elemental analysis. DSD was used as an amine curing agent, and the transparent, tensile strength-enhanced epoxy resins named EP–DSD were prepared via thermal curing reactions among the diglycidyl ether of bisphenol A (DGEBA), 4,4′-diaminodiphenylmethane (DDM), and DSD. The flame-retardancy of composites was studied by the limiting oxygen index (LOI) and UL-94 test. The LOI values of EP–DSD composites increased from 30.7% for a content of 3 wt % to 35.4% for a content of 9 wt %. When the content of DSD reached 6 wt %, a V-0 rating under the UL-94 vertical test was achieved. SEM photographs of char residues after the UL-94 test indicate that an intumescent and tight char layer with a porous structure inside was formed. The TGA results revealed that EP–DSD thermosets decomposed ahead of time. The graphitization degree of the residual chars was also investigated by laser Raman spectroscopy. The measurement of tensile strength at breaking point shows that the loading of DSD increases the tensile strength of epoxy thermosets. Py-GC/MS analysis shows the presence of phosphorus fragments released during EP–DSD thermal decomposition, which could act as free radical inhibitors in the gas phase. Owing to the promotion of the formation of intumescent and compact char residues in the condensed phase and nonflammable phosphorus fragments formed from the decomposition of DOPO groups, EP–DSD composites displayed obvious flame-retardancy.

## 1. Introduction

Epoxy resins are used in a broad variety of fields, such as electrical, electronics, and adhesive, as well as casting applications and construction because of their high adhesion, outstanding electrical and mechanical properties, and good alkali and moisture resistance [1,2,3]. However, the flammability and low transparency of epoxy resin composites are major disadvantages in their application, especially when used in circuit boards and coatings [4,5]. Several approaches have been utilized to enhance the thermal properties of epoxy resins, the most widely used method of which is the addition of flame-retardant additives, such as halogenated compounds, nitrogenous compounds, organophosphorus compounds, and inorganic materials [6,7,8,9].

Nanocomposites have taken a particular place in the preparation of transparent and flame-retardant coatings due to their thermal, light transmittance, and mechanical properties [10,11]. Morteza et al. prepared a new class of transparent epoxy-based nanocomposite coatings containing starch-modified nano-zinc oxide (ZnO–St), which can be applied as top-coats because of their transparency. They studied the curing behaviors of epoxy and transparent nanocomposites containing ZnO and ZnO–St, and found that the ZnO–St prolonged curing and increased the amount of heat release because of its autocatalytic effect [12]. Hadi et al. developed two kinds of layered double hydroxides (LDHs) and prepared the transparent nanocomposite coatings with sodium dodecylbenzene sulfonate (SDBS)-modified LDHs, Mg–Al and Zn–Al LDHs. Cure kinetics and viscoelastic behavior were studied. Network formation in the presence of SDBS-modified Zn–Al LDH nanoplatelets was considered to play an important role in the enhancement of epoxy ring-opening [13]. However, the flame retardancy of these nanocomposites, such as the limiting oxygen index (LOI) and UL-94 test, have not frequently been mentioned.

Due to the demand for environmentally friendly flame retardants (FRs) [14,15], organophosphorus compounds have been considered as dependable FR alternatives because of their good flame retardancy without the generation of lots of toxic smoke and corrosive gases [16]. While heating organophosphorus flame-retardant systems, an intumescent char layer is generated, acting as a physical isolation, which blocks heat transfer from the flame to the materials [17,18]. When combined with a nitrogen element, an obvious improvement in the flame-retardant efficiency is produced due to a phosphorus–nitrogen synergistic effect [19,20].

DOPO (9,10-dihydro-9-oxa-10-phosphaphenanthrene-10-oxide) has been frequently used as starting material for the synthesis of organophosphorus compounds. As a phosphorus element supplier, it can react with various electron-deficient compounds, such as unsaturated nitrogenous compounds, by the addition reaction of P–H, to produce various DOPO-based derivatives, and can then form phosphorus–nitrogen or phosphorus–silicon synergism [8,21,22]. There are lots of works reporting on the molecular design and synthesis of flame-retardant additives based on DOPO, as well as relevant flame-retardant epoxy resin composites [23,24]. The DOPO derivatives have proved to be excellent FRs due to their versatile flame extinguishing behavior in both the gas phase and condensed phase. In particular, for an intumescent flame-retardant system containing phosphorus and nitrogen, it can not only form an intumescent residue char, which works as a barrier to inhibit heat reflux in the condensed phase, but can also release many phosphorus fragments, which restrain the radical reaction in the gas phase [25,26,27]. However, these phosphorus-containing epoxy resin composites have some disadvantages, such as a low transparency, decreased mechanical properties, and a higher added ratio of DOPO-based derivatives due to their low phosphorus content, leading to a low number of applications [28,29]. This is necessary, not only to enhance the flame-retardant performance, but also to maintain its mechanical properties and transparency for coating. Yang et al. employed a hydroxyl-functionalized hyperbranched polymer (H30) to improve the mechanical properties of a diglycidyl ether of bisphenol A (DGEBA) epoxy resin. They found that the tensile strength was improved by adding a proper content of H30, and the glass transition temperature (*T*_g_) was not reduced by the addition of H30 in appropriate amounts [30]. Increasing the three-dimensional epoxy networks contributed to improvement of the tensile strength. As a nitrogen element supplier, 4,4′-diaminodiphenyl sulfone (DDS) is an easily available amine curing agent and compatible with DGEBA epoxy resin. When combined with phosphorus-containing compounds and reactive groups such as –OH, it can construct a phosphorus–nitrogen synergism with a multidimensional curing reaction.

In this work, one polyfunctional DOPO derivative defined as DSD was successfully synthesized and used as an amine curing agent to improve the flame-retardancy and tensile strength of DGEBA epoxy resins. Its chemical structure was well-characterized by FTIR–ATR, ^1^H NMR, and ^31^P NMR. The flame-retardant EP–DSD composites were prepared, and their flame-retardant properties were studied by the limiting oxygen index (LOI) and UL-94 test. Thermal decomposition, combustion behaviors, the structure of char residues, and the mechanical behaviors of these prepared thermosets were also investigated.

## 2. Materials and Methods

### 2.1. Materials

DOPO was supplied by Shanghai Nuotai Chemical Technology Reagent Co., Ltd. (Shanghai, China) and recrystallized in ethanol before use. DDS (4,4′-diaminodiphenyl sulfone) and salicylaldehyde were purchased from Aladdin Industrial Co., Ltd. (Shanghai, China). Tetrahydrofuran (THF) and 4,4′-diaminodiphenylmethane (DDM) were purchased from Sinopharm Chemical Reagent Co., Ltd. (Shanghai, China). Diglycidyl ether of bisphenol A (DGEBA, commercial name: E-44) was provided by Kunshan Lvxun Chemical Co., Ltd. (Kunshan, China).

### 2.2. Synthesis of DSD

**Scheme 1:**
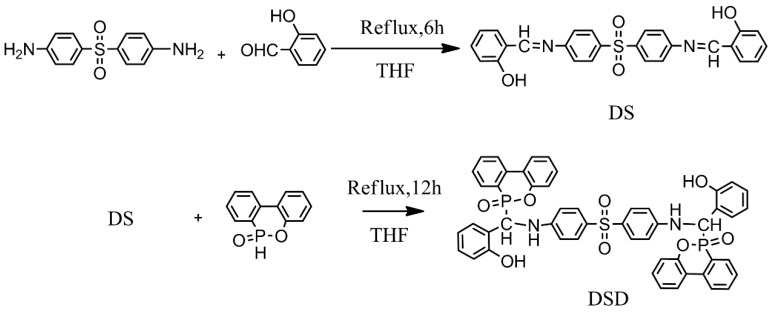
Synthetic routes of the intermediate imine (DS) and a 9,10-dihydro-9-oxa-10-phosphaphenanthrene-10-oxide (DOPO)-based flame retardant (DSD).

The synthetic routes of DSD are illustrated in Scheme 1. 

The intermediate imine coded as DS was synthesized by a typical condensation reaction; 4,4′-diaminodiphenyl sulfone (0.04 mol, 9.92 g) and THF 100 mL were fed into a 250 mL three-necked flask equipped with a magnetic stirrer and a reflux condenser, and the mixture was then heated to 40 °C. Subsequently, salicylaldehyde (0.08 mol, 9.77 g) dissolved in THF (60 mL) was added dropwise for 30 min under the protection of nitrogen. After this, the solution was heated to reflux temperature and maintained for 6 h. After cooling, the orange precipitate was filtered, washed with THF thoroughly, and dried at 60 °C for 18 h in a vacuum oven (17.62 g, yield: 96.5%).

The DSD was synthesized by an addition reaction. DS (0.02 mol, 9.13 g) and THF (80 mL) were added to a three-necked flask (250 mL) equipped with a thermometer, a reflux condenser, and a stirrer. Under the protection of nitrogen, DOPO (0.04 mol, 8.64 g) dissolved in THF (80 mL) was added for 1 h. With stirring, the reaction solution was heated and maintained at reflux temperature for 12 h. Then, the reaction solution was cooled, filtered by vacuum filtration, washed thoroughly with anhydrous alcohol and distilled water, and dried at 100 °C for 24 h until reaching a constant weight. A white solid was obtained (16.97 g, yield: 95.3%).

### 2.3. Preparation of Flame-Retardant Epoxy Resin

Flame-retardant epoxy resin was prepared via thermal curing reactions among DGEBA, DDM, and DSD. The formulations of modified epoxy resin are listed in Table 1, where the sum amounts of phenolic and amine protons are equal to the number of epoxy groups in DGEBA. DSD and DDM were blended at 90 °C for 30 min under a mechanical stirrer. When the mixture became transparent and uniform, the DGEBA was introduced and mixed for 5 min under a vacuum pump. Then, the mixture was poured into PTFE molds and cured at 120 °C for 2 h, followed by heat maintenance at 160 °C for another 2 h. Finally, the modified epoxy resin samples of a fixed size were cooled to room temperature slowly. The neat epoxy resin samples were also prepared under similar processing conditions, but without DSD. 

### 2.4. Characterization

The chemical structure of DSD was characterized by FTIR–ATR using a PerkinElmer Spectrum 2 (PerkinElmer, Akron, OH, USA), equipped with an attenuated total reflectance (ATR) accessory.

^1^H-NMR and ^31^P-NMR were performed on an Avance spectrometer (Bruker, Rheinstetten, Germany) at room temperature, and DMSO-d_6_ was used as the solvent.

TGA was performed on a TG 209 F1 thermal analyzer (NETZSCH, Selb, Germany), both in nitrogen and air atmosphere, with an operating temperature of 30 to 800 °C and heating rate of 10 °C/min.

Py-GC/MS was conducted on a Frontier PY-2020iD pyrolyzer connected to a Shimadzu GC–MS QP-2010 Ultra (SHIMADZU, Kyoto, Japan). The chromatographic separation was carried out in a capillary column (HP DB-5MS, 30 m length, 0.25 mm diameter, 0.25 mm thickness, Agilent, Valtbrone, Germany), and the carrier gas (helium) flow rate was 1 mL/min. The temperature of the chromatographic column was firstly held for 3 min at 40 °C, then heated to 300 °C at the rate of 15 °C/min, and finally kept at 300 °C for 10 min.

The limiting oxygen index (LOI) was measured on an oxygen index flammability gauge (ATS FAAR, Milan, Italy) based on ASTM D2863. The specimens were 130 mm in length, 6.5 mm in width, and 3 mm in thickness.

The UL-94 test was carried out according to the UL-94 standard, with specimen sizes of 130, 12.7, and 3 mm.

SEM was used to investigate the internal and external morphology of residue chars, which showed a raised appearance in the UL-94 test and was carried out on a TM-1000 scanning electron microscope (Hitachi, Tokyo, Japan), with 300 times magnification. The residue chars were stripped off by a needle and treated with gold sputtering on a copper station.

Raman spectra measurement was implemented on an SPEX-1430 laser Raman spectrometer (SPEX, Metuchen, NJ, USA), with an argon laser and a size of 633 nm.

The tensile strength was tested on an H10K-S material universal testing machine (Tiniius Olsen, Philadelphia, PA, USA), according to ASTM D638-08, at a stretching speed of 5 mm/min. The specimens were 75 mm long, 10 mm wide, and 2.1 mm thick, with a gage length of 30 mm. The results were averaged from eight tests.

## 3. Results

### 3.1. Characterization of DSD

The synthesis route of DSD is illustrated in Scheme 1. The intermediate DS was synthesized through aldimine condensation between DDS and salicylaldehyde. DSD was prepared from the addition reaction between the C=N groups of DS and the P–H groups of DOPO. The structure of DSD was characterized by FTIR and NMR.

**Figure 1:**
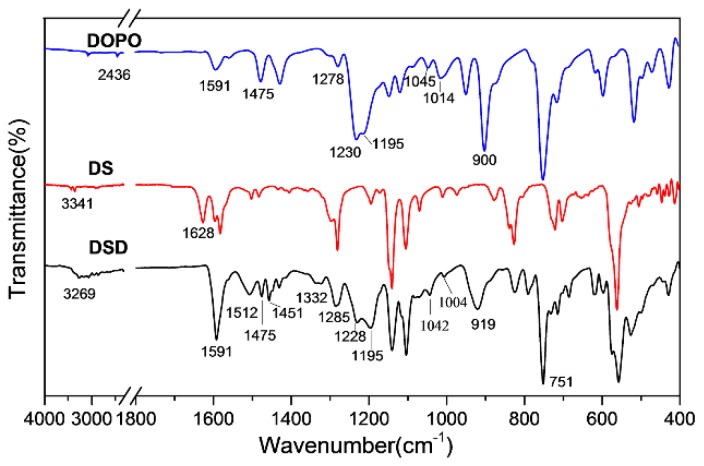
FTIR spectra of DOPO, DS, and DSD.

The FTIR spectra of DOPO and DSD are shown in Figure 1. In the FTIR spectra of DOPO, the absorption peak at 2436 cm^−1^ was assigned to the stretching vibration of P–H. The peaks at 1591 and 1475 cm^−1^ were attributed to the stretching vibrations of P–C_Ar_, and stretching vibration peaks of 1278 and 1230 cm^−1^ (P=O), 1045 and 1014 cm ^–1^ (P–O–C), and 1195 and 900 cm^−1^ (P–O–C_Ar_) could be observed. As for DS FTIR spectra, the peak at 1628 cm^−1^ could be attributed to the stretching vibrations of C=N, suggesting that DS was synthesized successfully. In the FTIR spectra of DSD, the absorption peaks were detected at 3269 and 1512 cm^−1^ (N–H), 1332 cm^−1^ (C–N), 1591 and 1475 cm^−1^ (P–C_Ar_), 1285 and 1228 cm^−1^ (P=O), 1042 and 1004 cm^−1^ (P–O–C), and 1195 and 919 cm^−1^ (P–O–C_Ar_). In addition, the peak of P–H, which was observed at 2436 cm^−1^ in DOPO, was not detected in the spectrum of DSD. This phenomenon means that the –P–H bond in the molecule of DOPO completely participated in the additive reaction with the –C=N– bond in DS. 

^1^H-NMR and ^31^P-NMR were conducted on a Bruker Avance spectrometer (400 MHz) at room temperature, using DMSO-*d*_6_ as the solvent. ^1^H-NMR (DMSO-*d*_6_, δ, ppm): 5.22–5.32 (2 H, P–C–H); 5.37–5.48 (2 H, –NH–); 6.44–8.30 (16 H, Ar–H); 9.48–9.73 (2 H, –OH).

**Figure 2:**
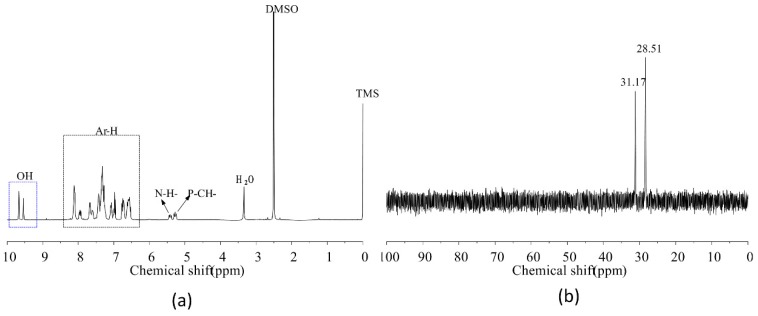
^1^H-NMR (**a**) and ^31^P-NMR(**b**) spectra of DSD.

The ^31^P-NMR spectrum of DSD is presented in Figure 2. Obviously, there was a double-peak between 28.51 and 31.17 ppm, corresponding to the two phosphorus atoms in DSD. It was considered that the bulky sulphone and rigid DOPO group existed in the molecular structure of DSD, and their steric hindrance effects caused the unequal phosphorus peaks [2,31].

Elemental analysis was also executed to confirm the chemical structure of DSD. Elemental analysis values were as follows: C, 66.29%; H, 4.60%; N, 2.99%; and P, 7.04%. Calculated values were as follows: C, 67.56%; H, 4.31%; N, 3.15%; and P, 6.97%. It is clear that the elemental analysis values of DSD were in accordance with the calculated values. All of these results indicated that the DSD was synthesized successfully.

### 3.2. Flame-Retardant Properties of Epoxy Thermosets

The flame-retardant properties of EP and EP–DSD thermosets were evaluated by the LOI and UL-94 test. The relevant data from these tests are listed in Table 2.

As shown in Table 2, the LOI value of neat EP was only 24.8%, which continued burning with fire drippings after the first ignition, and the drippings could ignite the cotton. In contrast, the EP/DSD composites showed an improved flame-retardancy, and the LOI values of the EP–DSD thermosets obviously increased from 30.7% to 35.4% with the increasing of the DSD content from 0 to 9 wt %. When the loadings of DSD were more than 6 wt %, the composites passed V-0 ratings in UL-94 tests. These results demonstrate that DSD significantly improved the flame-retardancy of epoxy thermosets with a high efficiency.

**Figure 3:**
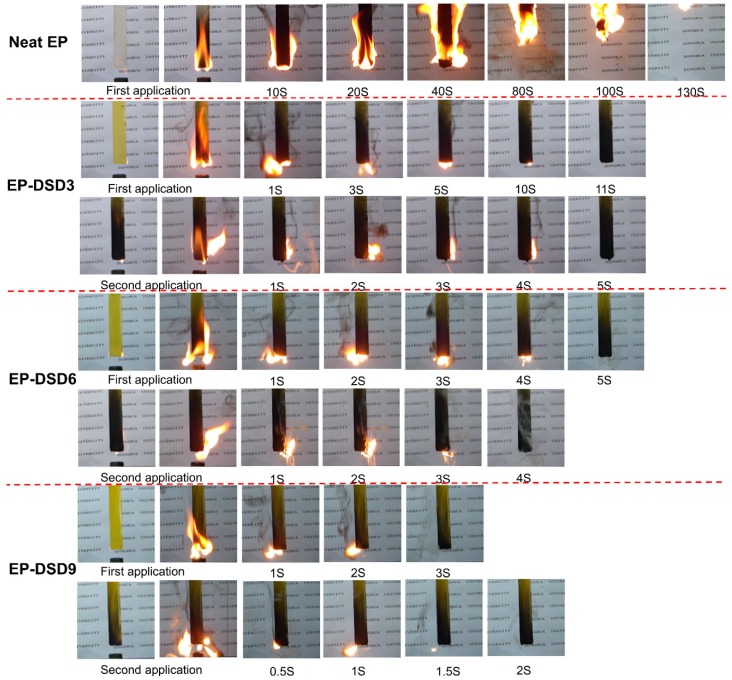
Digital photos of EP thermosets during the UL-94 test.

The combustion of neat EP and EP–DSD thermosets during the UL-94 test was recorded by a digital camera. The relevant screenshots are shown in Figure 3. Obviously, the neat EP combusted vigorously with flaming drips after the first ignition, which showed that it was highly flammable. In regard to EP–DSD thermosets, the combustion behaviors differed from those of neat EP, and they all extinguished spontaneously. With the increasing of the DSD content, the burning times after the first and second ignition decreased gradually. Moreover, the pyrolytic gases were found to be continuously ejected from the interiors of samples during combustion, and this ejection of gas could even blow the flame out at times. Some studies have also reported this phenomenon, called “blowing-out”, which helps to reduce the combustion time and improve the flame-retardancy of thermosets [32].

### 3.3. Morphology and Structure of Residual Char after the UL-94 Test

**Figure 4:**
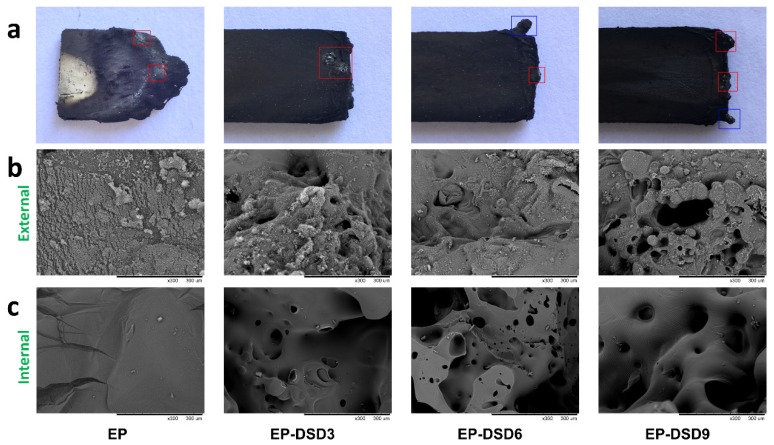
Digital photos and SEM photographs of neat EP and EP–DSD thermoset char residues after the UL-94 test. (**a**) Digital photos of char residues after UL-94 test, (**b**) SEM photographs of external char residues marked with red box, (**c**) SEM photographs of internal char residues marked with red box. Red box: Char residues was observed by SEM. Blue box: Raised stomates which found on char residues.

To know more about the combustion behavior of neat EP and EP–DSD thermosets in the UL-94 test, the photographs recorded by a digital camera and SEM micrographs of char residues are shown in Figure 4.

As presented in Figure 4a, neat EP possessed a loose and cotton-like char layer with distinct cracks after the UL-94 test. Unlike neat EP, the residual char of EP–DSD thermosets maintained a cuboid morphology, which the UL-94 test required for samples, and the char layer of EP–DSD thermosets was relatively tight and smooth. Furthermore, for EP–DSD thermosets, it should be noted that there were several raised stomates marked with the blue box on the surface of residual char, where pyrolytic gases were found to be ejected during the UL-94 test. It is possible that the loading of DSD enhanced the strength of the char layer, and the strength was strong enough to maintain the shape of raised stomates due to the high internal pressure caused by the accumulation of pyrolytic gases during combustion.

The SEM micrographs of char residues corresponding to the red box in Figure 4a are shown in Figure 4b,c. It is clear that the char residue of neat EP displayed a discontinuous morphology with some cracks. For EP–DSD thermosets, the external char layer showed a tight and intumescent morphology with small holes, and the internal char layer possessed a smooth morphology with some honeycombed cavities. It is presumable that this tight and intumescent char layer with a honeycombed cavity inside acted as an insulation layer, and could block the fluxion of air and heat transfer during combustion [33].

In addition, this insulation layer could also prevent the pyrolytic gases generated inside the samples from escaping. As the amount of pyrolytic gases increased, the inside pressure increased. Under sufficient pressure, the pyrolytic gases broke through the char layer and blew out the flame due to the quenching effect of phosphorus radicals and the diluting effect of nonflammable gases. All of these effects mean that the EP–DSD thermosets achieved a short after-flame time.

### 3.4. Morphology of Residual Char after Thermal Degradation under Nitrogen Atmosphere

**Figure 5:**
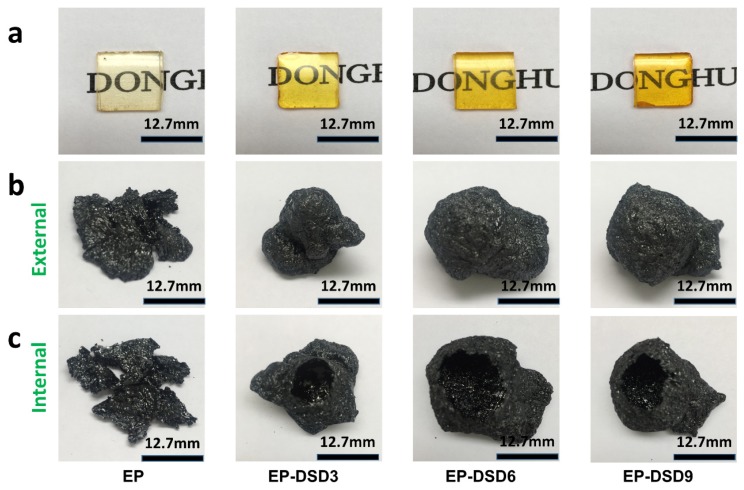
Digital photos of epoxy thermosets before (**a**) and after (**b**,**c**) thermal degradation in nitrogen.

To learn more about the morphology of the char layer, neat EP and EP–DSD thermosets (12.7 mm × 12.7 mm × 3.0 mm) were heated to 600 °C at a heating rate of 10 °C/min under nitrogen atmosphere in a muffle furnace, and naturally cooled to room temperature. A digital photograph of epoxy thermosets before and after thermal degradation is presented in Figure 5.

Figure 5a presents digital photos of neat EP and EP–DSD thermosets, and all EP thermosets exhibited a transparent appearance. As a part of the curing agent, DSD could participate in the curing action with DGEBA and DDM. After curing, EP–DSD thermosets could be considered as whole in the chemical structure. Unlike some inorganic materials, such as montmorillonite (MMT) and nanofillers, DSD is not dispersed in EP, so the presence of stacks and phase separation can be avoided in EP–DSD thermosets [11,34]. He et al. have reported a flame-retardant EP/MMT composite with an opaque and dark brown appearance, which resulted from the visible MMT particles in the EP composite [33]. In addition, when heated to melting in the preparation of EP–DSD composites, the mixture of DDM, DSD, and DGEBA also presented a transparent liquid. It could be deduced that the absence of stacks derived from completely participating in the curing action and good compatibility with the EP matrix, resulting in the transparent appearance of EP–DSD thermosets [35].

As shown in Figure 5b,c, the neat EP presented a fragile and lamellar char layer, while EP–DSD thermosets showed an intumescent and three-dimensional structural morphology. The surface char layers were carefully cut open by a razor blade, showing that the interior of residual chars for EP–DSD thermosets was hollow and presented a huge cavity, which could gather a certain amount of pyrolytic gases. This indicates that the loading of DSD could enhance the char layer cohesion, which acted like a more efficient physical barrier to pyrolysis gas mass transfer. When heated, with the increasing amount of pyrolytic gases generated and accumulated under the char layer, an intumescent morphology with a porous structure inside was finally exhibited.

**Figure 6:**
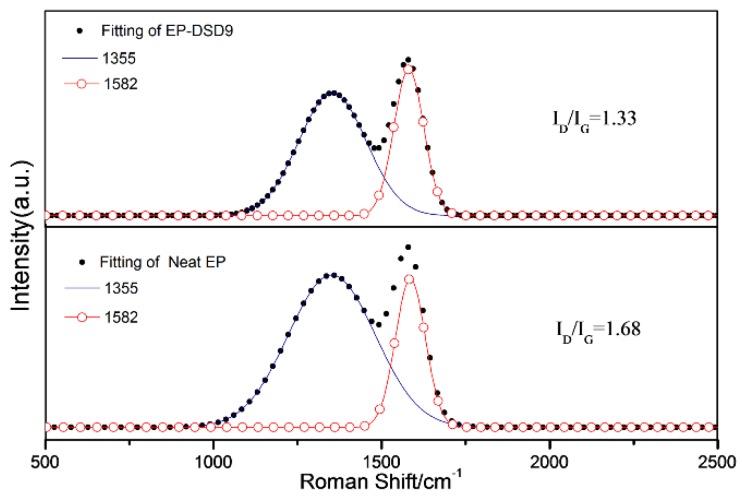
Raman spectra of neat EP and EP–DSD9.

For further investigation, the residual chars for neat EP and EP–DSD9 thermosets after thermal degradation under nitrogen atmosphere were examined by laser Raman spectroscopy. The corresponding Raman spectra of residue char are presented in Figure 6. Both of the two spectra displayed two peaks at 1355 cm^−1^ (D band) and 1582 cm^−1^ (G band). Generally, the D band corresponds to carbons in a disorder arrangement, while the G band relates to carbons in graphite layers [9,36,37]. The intensity of D and G bands was represented by the relevant peak area, and the ratio of the intensity of D and G bands (*I*_D_/*I*_G_) of neat EP and EP–DSD9 thermosets was calculated by the ratio of peat areas. As shown in Figure 6, the I_D_/I_G_ values of neat EP and EP–DSD9 thermosets were 1.68 and 1.33, respectively. This means that the residual char of EP–DSD9 thermosets possessed a higher graphitization degree, and thus had a higher heat stability.

### 3.5. Thermal Stability of EP and EP/DSD Thermosets

**Figure 7:**
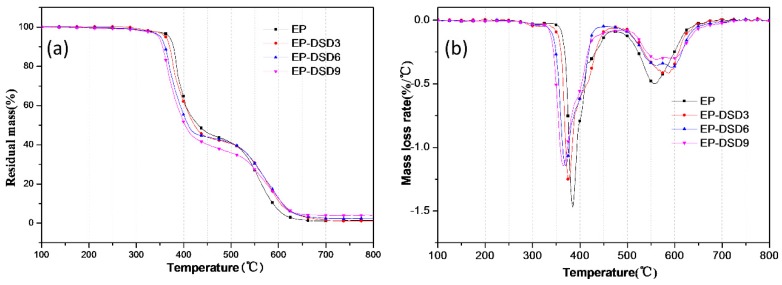
TG (**a**) and DTG (**b**) curves of epoxy thermosets under air atmosphere.

**Figure 8:**
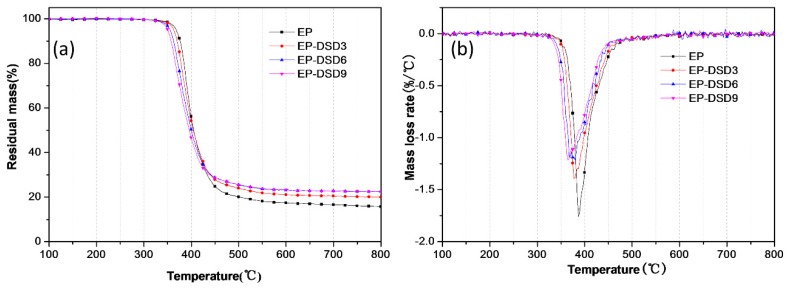
TG (**a**) and DTG (**b**) curves of neat EP and EP–DSD thermosets under nitrogen atmosphere.

The thermal stability of neat EP and EP/DSD thermosets was investigated by TGA under air and nitrogen atmosphere, and the TG and DTG curves of epoxy thermosets are shown in Figure 7 and Figure 8, respectively. The values of the initial degradation temperature for 5% weight loss (*T*_5%_), the maximum weightlessness rate (*V*_max_), the temperature at *V*_max_ (*T*_max_), and the char yield at *T*_max_ and 700 °C (*CY*_Tmax_ and *CY*_700_) are listed in Table 3 and Table 4.

As Figure 7 shows, there were two rapid descents in the TG curve, which corresponded to the two peaks in the DTG curve. These indicated that there were two weight-loss processes in the thermal degradation of all EP thermosets under air atmosphere. However, it was clear that the TG and DTG curves of EP–DSD thermosets were different from those of neat EP. Although the loading of DSD did not change the two-stage thermal degradation of EP thermosets under air atmosphere, it advanced the decomposition time. As shown in Table 3, compared with neat EP, the loading of DSD resulted in a lower *T*_5%_ for EP/DSD thermosets, and it decreased from 369.7 °C for neat EP to 346.4 °C for EP–DSD9. Besides, with the increasing of DSD content, higher char yields at *T*_max_ and 700 °C could be observed when compared with neat EP. This suggests that the introduction of DSD could reduce the weight loss and promote the formation of char residues, and hence improve the thermal stability.

Under nitrogen atmosphere, the TG curves of all EP thermosets displayed a single decomposition process, as shown in Figure 8, which corresponded to the only peak in the DTG curve. With the increase of DSD content from 0 wt % to 9 wt %, the char residue at 700 °C was gradually increased from 16.58% to 23.45%. Similar to the results in air atmosphere, with the dosage of DSD, the char residual weights were much larger than those of neat EP, proving that an enhanced performance was exhibited by char forming under nitrogen atmosphere. In addition, the *T*_5%_, *T*_max_, and *V*_max_ of EP–DSD thermosets were lower than those of neat EP with the increasing content of DSD.

In summary, EP–DSD thermosets possessed a higher char yield than neat EP in both air and nitrogen atmosphere. The loading of DSD lowered the *T*_5%_, *T*_max_, and *V*_max_ of thermosets. These changes indicated that the loading of DSD contributed to char formation in combustion, which means a better enhancement of the flame-retardancy of epoxy thermosets. 

### 3.6. Py-GC/MS Analysis of DSD, EP, and EP–DSD Thermosets

**Figure 9:**
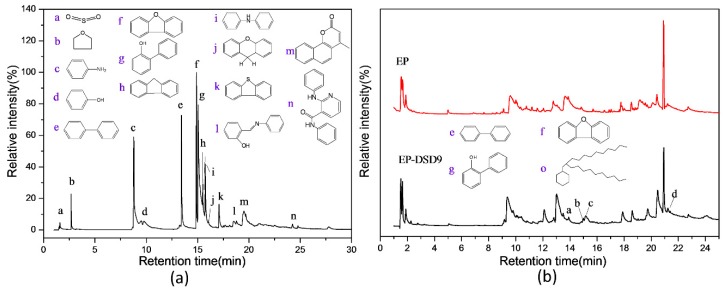
Pyrograms of DSD (**a**), and neat EP and EP–DSD (**b**) thermosets.

As shown in Figure 9, there were 14 kinds of pyrolysis products for DSD. However, the main pyrolytic products of DSD were benzenamine (c, relative area: 21.98%), diphenyl (e, relative area: 8.29%), dibenzofuran (f, relative area: 11.18%), o-phenylphenol (g, relative area: 36.07%), and fluorene (h, relative area: 1.17%). In consideration of the molecular structure and their relative area, it is reasonable that the breaking of C–N and C–P in DSD resulted in the main pyrolytic products above, and the diphenyl (e), dibenzofuran (f), and *o*-phenylphenol (g) were decomposition products of DOPO-groups. This suggest that phosphorus fragments existed in the gas phase [38].

**Figure 10:**
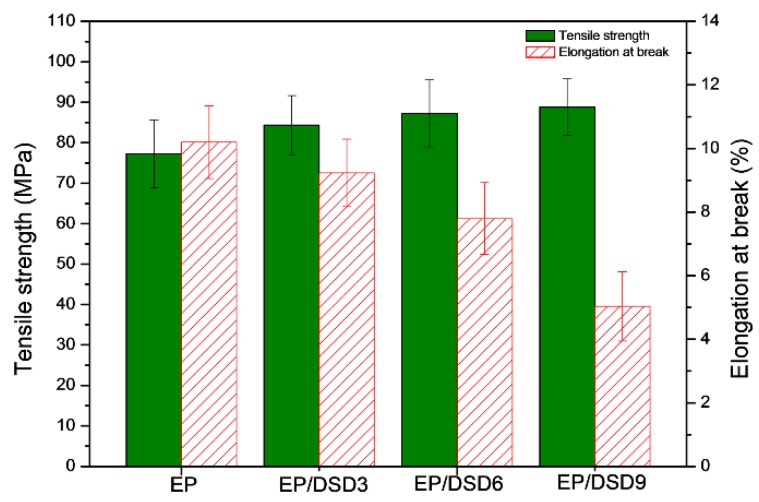
Strength and elongation at the breaking point of epoxy thermosets.

Figure 10 shows the pyrograms of neat EP and EP–DSD thermosets; they were similar, but the relative area of EP–DSD thermosets was large. It should be noted that diphenyl (e), dibenzofuran (f), and o-phenylphenol (g) were also observed in EP–DSD pyrograms, which related to decomposition of the DOPO-groups, indicating that phosphorus fragments released in the decomposition of EP–DSD thermosets could inhibit the free radical reaction in the flame in the gas phase.

### 3.7. Mechanical Properties of Neat EP and EP–DSD Thermosets

When used as a curing agent, the influence of DSD on tensile properties was investigated by measurement of the tensile strength and elongation. The results are exhibited in Figure 10. Neat EP displayed outstanding tensile properties, with a tensile strength of 77.29 MPa and elongation of 8.39% at breaking point. The addition of DSD improved the tensile strength of EP–DSD composites from 84.37 MPa for EP–DSD3 to 88.83 MPa for EP–DSD9. Cross-linking degree values were calculated and are listed in Table 5. They increased from 1958 mol/m^3^ for neat EP to 2105 mol/m^3^ for EP–DSD9, and thus raised the *T*_g_ from 171.2 to 176.5 °C, complying with the increasing of tensile strength. This could be attributed to the larger cross-linked network structure, which was formed by the cross-linking of OH and –NH– groups in DSD with DGEBA and DDM. However, the cross-linked network structure and the rigid DOPO group constrained the molecular chain motion [39], and the elongation of EP–DSD thermosets decreased with the increasing dosage of DSD. 

## 4. Conclusions

In this study, a novel DOPO-based flame retardant (DSD) was successfully synthesized, and its chemical structure was confirmed by FTIR, ^1^H-NMR, and ^31^P-NMR. TGA analysis showed that the loading of DSD improved the thermal stability of EP composites, lowered the *T*_5%_ and *T*_max_ values of EP thermosets, and increased the char residue to 3.98% and 23.45% under air and nitrogen atmosphere, respectively. The results from the LOI and Ul-94 test releveled that the addition of DSD improved the flame retardancy of EP composites. Digital photos and SEM micrographs illustrated that a compact and stable intumescent char layer with a honeycombed cavity inside was formed after combustion, which was responsible for the enhanced flame-retardant properties. Furthermore, the *I*_D_/*I*_G_ values of neat EP and EP–DSD9 thermosets, which were calculated according to Raman spectra, indicate that the residual char of EP–DSD9 thermosets possesses a higher graphitization degree, and thus has a higher heat stability. Py-GC/MS analysis showed that phosphorus fragments were released in the decomposition of EP–DSD thermosets, which could inhibit the free radical reaction in the gas phase. The formation of a larger cross-linked network structure due to the cross-linking of the OH and –NH– group in DSD with DGEBA and DDM resulted in an enhancement of the tensile strength for EP–DSD composites.

## Figures and Tables

**Table 1:** The formulas of EP–DSD composites.

Samples	Composition (g)			DSD (wt %)	P (wt %)	N (wt %)
	DGEBA	DDM	DSD			
EP0	100	22	0	0	0	2.54
EP–DSD3	100	21.16	3.75	3	0.21	2.49
EP–DSD6	100	19.38	7.62	6	0.42	2.35
EP–DSD9	100	19.37	11.81	9	0.63	2.37

**Table 2:** Flame-retardancy of EP and EP–DSD thermosets.

Samples	LOI (%)	UL-94, 3.0 mm bar			
		*t*_1_ + *t*_2_^a^ (s)	Ignition of Cotton	Dripping	Rating
EP	24.8	NR ^b^	Yes	Yes	No rating
EP–DSD3	30.7	11.5 + 5.8	NO	NO	V-1
EP–DSD6	34.2	5.5 + 4.2	NO	NO	V-0
EP–DSD9	35.4	3.1 + 2.3	NO	NO	V-0

^a^*t*_1_ and *t*_2_: average after-flame times after the first and the second flame application. ^b^ No record due to burn out.

**Table 3:** TGA data of epoxy thermosets under air atmosphere.

Sample	*T*_5%_ (°C)	*T*_max_ (°C)	*CY*_Tmax_ (%)	*V*_max_ (%/°C)	*CY*_700_ (%)
EP	369.7	384.9	79.84	−1.47	1.21
EP–DSD3	361.3	375.9	79.92	−1.25	1.54
EP–DSD6	351.1	369.7	80.28	−1.14	2.49
EP–DSD9	346.4	364.5	82.97	−1.14	3.98

**Table 4:** Data of neat EP and EP–DSD thermosets under nitrogen atmosphere.

Sample	*T*_5%_ (°C)	*T*_max_ (°C)	*CY*_Tmax_ (%)	*V*_max_ (%/°C)	*CY*_700_ (%)
EP	367.2	387.2	75.78	−1.76	16.58
EP–DSD3	362.2	377.2	79.49	−1.39	20.48
EP–DSD6	354.6	372.1	81.73	−1.25	22.74
EP–DSD9	349.7	364.7	82.13	−1.21	22.87

**Table 5:** Glass transition temperature (*T*_g_) and cross-linking degree values of neat EP and EP–DSD thermosets.

Sample	*T*_g_ (°C)	*ν*_e_ (mol/m^3^)
EP	171.2	1958
EP–DSD3	173.6	2015
EP–DSD6	174.9	2067
EP–DSD9	176.5	2105
